# A magnetic X-band frequency microwave nanoabsorbent made of iron oxide/halloysite nanostructures combined with polystyrene†

**DOI:** 10.1039/d2ra08339f

**Published:** 2023-02-27

**Authors:** Diana Fallah Jelodar, Mojtaba Rouhi, Reza Taheri-Ledari, Zoleikha Hajizadeh, Ali Maleki

**Affiliations:** a Catalysts and Organic Synthesis Research Laboratory, Department of Chemistry, Iran University of Science and Technology Tehran 16846-13114 Iran maleki@iust.ac.ir +98-21-73021584 +98-21-73228313; b Department of Physics, Iran University of Science and Technology Tehran 16846-13114 Iran

## Abstract

A novel nanocomposite has been designed and fabricated through an *in situ* polymerization process, based on iron oxide nanoparticles (Fe_3_O_4_ NPs), halloysite nanotubes (HNTs), and polystyrene (PS). The prepared nanocomposite (formulated as Fe_3_O_4_/HNT-PS) has been fully characterized through various methods, and its applicability in microwave absorption was investigated by using some single-layer and bilayer pellets containing nanocomposite and resin. The efficiency of the Fe_3_O_4_/HNT-PS composite with different weight ratios and pellets with the thickness of 3.0 and 4.0 mm were examined. Vector network analysis (VNA) revealed that the microwave (12 GHz) can be noticeably absorbed by Fe_3_O_4_/HNT-60% PS particles in a bilayer structure with 4.0 mm thickness and 85% resin of the pellets, resulting in a microwave absorption value of *ca.* −26.9 dB. The observed bandwidth (RL < −10 dB) was about 1.27 GHz, where *ca.* 95% of the radiated wave is absorbed. Ultimately, due to low-cost raw materials and high performance of the presented absorbent system, the Fe_3_O_4_/HNT-PS nanocomposite and the construction of the presented bilayer system can be subjected to further investigations to test and compare with other compounds for industrialization.

## Abstract 2

A magnetic hybrid nanoabsorbent made of iron oxide, halloysite and polystyrene is fabricated and utilized for the absorption of X-band microwaves.

## Graphical abstract

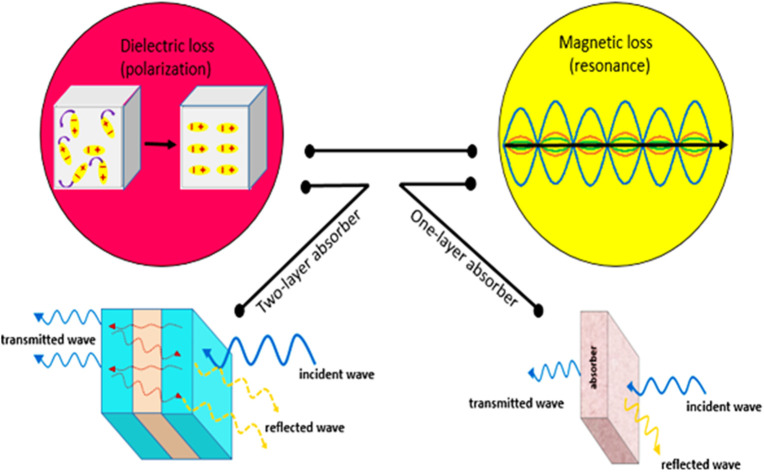

## 1. Introduction

With the passage of time and in accordance with the brilliant advances in energy technology, the problems facing human beings are becoming more complex. Today, extensive utilization of wireless devices such as telecommunication networks, radar and electronic equipment, has led researchers to pay more attention to electromagnetic interference (EMI).^1–4^ Hence, in order to reduce the dangers of EMI (especially microwave contamination), efficient absorbent systems are developed.^5–8^ As the most well-known species, coating materials including magnetic particles,^9^ carbon-based nanostructures,^10^ metal/carbon composites,^11^ and metal oxides,^12^ have shown great capability to absorb microwave energy and reduce their reflection and transmission in a certain frequency range depending on their exclusive electrical and magnetic characteristics.^13^ The most important electrical and magnetic mechanisms that are suggested for the microwave absorption are dielectric polarization and magnetic resonance of the absorbent system, respectively.^14^ The desirable results for magnetic or dielectric loss can be achieved by simultaneous application of magnetic and carbon-based materials.^15^ Also, improvement of microwave absorption and overlap of defects can be achieved by combining these materials.^16,17^ Some composite structures such as Fe–Fe_3_C-MWCNT and FeCo-CNTs are introduced for improving the absorption properties in a certain bandwidth.^18–21^

Metals with exclusive features such as good microwave absorption capability, high bandwidth values, low price and high temperature stability, are considered as the most efficient materials for EMI shielding purposes.^22,23^ However, their heavy weight and prone to corrosion lead researchers to look for better alternatives.^24–27^ Therefore, materials that include high bandwidth, lightness, low cost, thermal and chemical stabilities are highly considered for the EMI aims.^28–31^ Combination of magneto-electric materials with other species which include unique physicochemical properties can also be a good strategy for studying the EMI shielding.^32^ Among all species utilized in composite systems, polystyrene (PS) (as a non-conductive polymer^33^) has been utilized in the composite microwave absorbent system.^34^ PS is an appropriate agent for integration of the targeted components in the same composite system. As mentioned before it, the electric, magnetic and conduction loss are three fundamental parameters in the microwave absorption process.^14^ Utilization of the PS in the composite systems also limits the conditions to a couple modes; electric loss and magnetic loss. In the same line, the PS is selected in this research to lead the conditions to a true comparison between two other components involved in the composite system. In a pioneering work in the field of the absorbent composites, Heidari *et al.*^35^ studied electromagnetic wave absorption properties of ZnO, Fe_3_O_4_ and graphene oxide in a PS-based composite system containing 7 wt% graphene oxide, which resulted in a higher amount of loss (−7.2 dB at 7.15 GHz). Although, the PS seems to be a conductive agent due to the presence of benzene ring and carbon double bonds at its structure, it is not able to conduct electricity because of its high energy gap in comparison with the conventional conductive materials such as metals.^36^

As another suitable ingredient for designing a substantial microwave absorbent system, iron oxide nanoparticles (Fe_3_O_4_ NPs) can be mentioned.^37–40^ There are so many advantages for the use of Fe_3_O_4_ NPs, as follows; nontoxicity,^41^ biodegradability and biocompatibility,^42^ capability to be covalently functionalized,^43^ well structural stability,^44^ recyclability,^45^ low-cost and convenient preparation methods,^46^ tiny size (average diameter: 40 nm),^47^ and super-paramagnetic behavior.^48^ As well, the Fe_3_O_4_ NPs with an appropriate band gap value (*ca.* 2.5 eV) has shown great compatibility with the other active components in the microwave absorption process.^49^ As an instance, Huang *et al.* reported a strategy to easily prepare an iron-based magnetic carbon microtube nanocomposite (formulated as CNT/Fe_3_O_4_), which demonstrated a reflection loss of −40 dB at 10.64 GHz, and an effective absorbing bandwidth of 4 GHz with a thickness of 2.0 mm.^50^ As a suitable substrate for the composition of Fe_3_O_4_ NPs, the materials with alumina and silica network (like halloysite nanotubes, abbreviated as HNTs) are effective for microwave absorption, too.^51–53^ Due to the presence of silicon and especially aluminum, this type of materials is effective in increasing the microwave absorption capability.^54^ Moreover, its unique structure (mineral nanotubes with high surface area) would be appropriate for incorporation of the magnetite nanoparticles through a layer-by-layer manner, which is effective in increasing of the EMI.^55,56^

In studies and researches, all factors must be sequentially examined to achieve the desired results. In our previous projects,^37,53^ studies were accomplished on the effects of polypyrrole (as a conductive polymer), Fe_3_O_4_ magnetic nanoparticles, and HNTs as a mineral aluminosilicate. In current research, considering the mentioned three main mechanisms of microwave absorption (dielectric loss, magnetic loss, and conduction loss), the purpose is to specifically limit conduction loss by the use of non-conductive PS. Since HNT inherently includes high porosity, the PS polymer and Fe_3_O_4_ nanoparticles could be incorporated into the rolled tubular structure of the HNTs through a layer-by-layer pattern, which may increase the efficiency of the system.^37^ Also, the Fe_3_O_4_ magnetic nanoparticles have been inserted into the composite structure because they induce magnetic loss without any role in two other waves' absorption mechanisms.^14^ To investigate the effects of the various ratio of the involved ingredients on efficiency of the nanocomposite, two types of composite with different weight ratio of PS were prepared *via* the explained procedure; Fe_3_O_4_/HNT-60% PS and Fe_3_O_4_/HNT-72% PS. Also, the single-layer and bilayer structures were prepared and studied under the same conditions. Concisely, it was observed that a reflection loss value of −26.9 dB over the bilayer sample of Fe_3_O_4_/HNT-60% PS with 4.0 mm thickness and bandwidth (RL < −10 dB) was about 1.27 GHz, with a microwave absorption performance of 95%.

## 2. Experimental

### 2.1. Materials & equipment

#### 2.1.1. Preparations

All solvents and reagents including ammonia, styrene, and octanol were purchased from Merck and Sigma Aldrich without further purification. Also, the iron salts (FeCl_3_·6H_2_O and FeCl_2_·4H_2_O), benzoyl peroxide (BPO, 72% purity & synthesis-grade), and sodium dodecyl sulfate (SDS, 90% purity & technical-grade) were provided from Merck and used without further purification. Halloysite nanotubes (HNTs, Technical Grade, CAS:1332-58-7) was prepared from Sigma Aldrich.

#### 2.1.2. Characterizations

Fourier transform infrared (FTIR) spectra were recorded on a FTIR-8400 Shimadzu Spectrum Scanner, Japan. The size and morphology of the solid-sate particles were studied with a scanning electronic microscopy (FESEM) (KYKY EM8000f, China), with a Numerix DXP-X10P attached capable to perform energy-dispersive X-ray (EDX) analysis. The structural stability and thermal resistance of the samples were investigated by thermogravimetric analysis (TGA) over a Bahr-STA 504 instrument (USA). A vibrating-sample magnetometer (LBKFB model-magnetic kavir, Iran) was applied to determine the magnetic saturation of the prepared magnetic samples. The microwave absorption properties were measured by Vector Network Analysers (Agilent Technologies, E5071C, USA) in the X-band range, which as a spectrophotometer, can determine the amount of microwave absorption and its percentage through direct irradiation of the microwave to the samples in the form of tablet.

### 2.2. Preparation methods

#### 2.2.1. Preparation of Fe_3_O_4_/HNTs

The preparation of Fe_3_O_4_/halloysite nanotubes was carried out according to the co-deposition procedures given in the last reports.^57–59^ Firstly, in a round-bottom flask (500 mL), 0.1 g of HNTs was dispersed in 200 mL of distilled water, *via* ultrasonication in a cleaner bath (50 kHz, 100 W L^−1^). Next, 1.65 g (6.1 mmol) of FeCl_3_·6H_2_O and 0.430 g (2.1 mmol) of FeCl_2_·4H_2_O were added into the aqueous mixture of HNTs, and stirred for 30 min at 80 °C. Then, 20 mL of ammonia solution (25%) was added into the mixture through a dropwise manner. The final mixture was injected into a container with two inlets; one end of which was connected to a refrigerant containing N_2_ gas inlet, and the other end was poured through an ammonia-containing syringe. While addition of a drop of ammonia solution, black particles were immediately appeared confirming successful formation of Fe_3_O_4_ nanoparticles.^60–62^ After completion of the addition, the mixture was well homogenized *via* stirring for 45 min, at room temperature. The formed Fe_3_O_4_/HNTs particles were separated by holding a super-magnet at the end of container. Finally, the particles were washed several times with distilled water, and were dried at room temperature, for 24 hours.

#### 2.2.2. Preparation of Fe_3_O_4_/HNT-PS nanocomposite

For the synthesis of PS according to the instructions,^63^ in a round-bottom flask (250 mL), octanol (3.0 mL) was added to an aqueous solution of sodium dodecyl sulfate (SDS, 0.5 g, 1.7 mmol) in 150 mL of deionized (DI) water and stirred at room temperature. Then, 0.2 g of Fe_3_O_4_/HNTs was added into the solution, and the mixture was ultrasonicated for 30 min (50 kHz, 100 W L^−1^). Next, the flask was transferred into an ice bath, and equipped to a nitrogen inlet condenser condition. Afterward, styrene (5.0 g, 48.0 mmol) and benzoyl peroxide (BPO, 0.05 g, 0.2 mmol) were added into the mixture and the content was stirred for 30 min, at room temperature. At this stage, an *in situ* polymerization was performed by sonication (50 kHz, 100 W L^−1^) at 0 °C for 30 min, and then stirring in the oil bath at 80 °C for 4 h under the nitrogen flow. The excess of the surfactant and residual monomers were removed by washing the particles with methanol and water. Finally, the Fe_3_O_4_/HNT-PS particles were magnetically separated and dried at 60 °C, for 24 h. To investigate the effect of the various ratio of the involved ingredients on efficiency of the nanocomposite, two types of Fe_3_O_4_/HNT-PS composite with different weight ratio of styrene (wt%, based on TGA results) were prepared *via* the explained procedure; Fe_3_O_4_/HNT-60% PS and Fe_3_O_4_/HNT-72% PS. Because it was intended to observe the effect of the amount of PS, two different amounts of polymer were used for preparation of the magnetic nanocomposite.

### 2.3. Mechanism of microwave absorption

Electrical reagent for the amount of absorption known as electrical permittivity is represented by “*ε*” and is defined as the real and imaginary part defined by eqn (1), in which the imaginary part presents the amount of the energy lost:^35^

1 *ε*_r_ = *ε*′ − *iε*′′

The effective magnetic reagent for the absorption is also known as magnetic permeability. This value is represented by “*μ*” and defined by eqn (2), in which the imaginary part shows the amount of energy loss and the actual amount of absorption^35^

2 *μ*_r_ = *μ*′ − *iμ*′′

In addition, microwave absorbents with the properties such as low thickness, high bandwidth, high heat resistance, low density, reasonable price, chemical stability and the ability to cover the instrument, have the desired electrical and magnetic properties for the absorption process.^64,65^ The RL (reflection loss) of an electromagnetic wave is obtained when the incident waves collide in a perpendicular position to the surface of the absorbent material with a metal background (eqn (3)):3
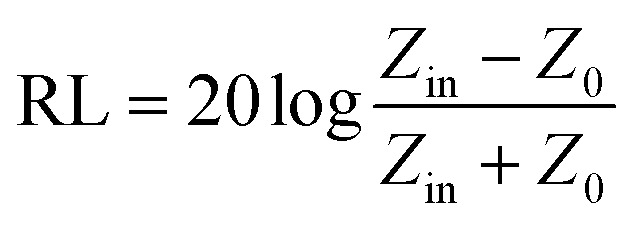
where, “*Z*_0_” is the characteristic impedance of free space and “*Z*_in_” is the input impedance at the intersection of matter and free space, which is expressed by eqn (4):4
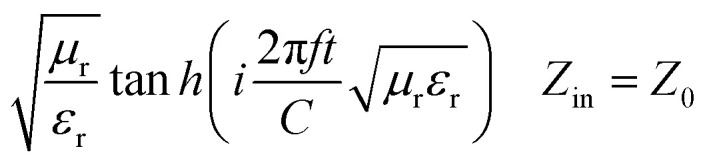
where, “*c*” is the electromagnetic wave velocity in the free space, “*f*” and “*t*” are frequency of the microwave and the thickness of the absorbent, respectively.^35^ The reflection loss of an absorbent is a function of following six characteristic parameters: *f* and *t*′′, *μ*′, *μ*′′, *ε*′′, *ε*.

**Scheme 1:**
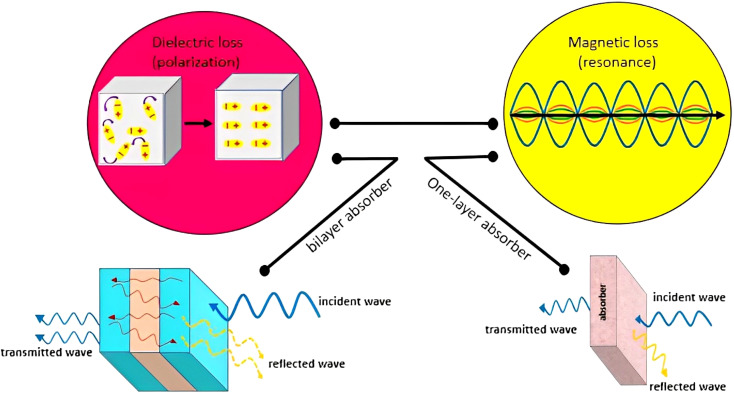
Schematic presentation of the mechanism of microwave absorption in single-layer and bilayer structures.

Scheme 1 shows the mechanism of microwave absorption as well as the way of radiation to the single-layer and bilayer microwave absorbent systems. The absorption mechanism includes the conditions of microwave absorption of dielectric loss and magnetic loss due to the use of PS which is a non-conductive polymer (thus conduction loss is quenched). Regarding the performance of radiation, it should be noticed that in a single-layer structure, the wave radiated to the absorbent system is absorbed, reflected and transmitted. While in the bilayer structure, in addition to the main reflection and transmission (due to the trapping the microwaves between the layers and reciprocating behavior), secondary reflection and transmission (red lines) also occur due to presence of resin as a dielectric. The single-layer absorbent has two thicknesses of 3.0 mm and 4.0 mm. It should be noted that the thickness of the resin used in the bilayer absorbent is 1.0 mm and the thickness of both sides of the absorbent is the same. It means that for the bilayer absorbent (thickness of 3.0 mm and 4.0 mm), the thickness of Fe_3_O_4_/HNT-PS magnetic nanocomposite layer is 1.0 mm and 1.5 mm, respectively. Indeed, the resin layer is not considered as a separate layer because it has very low microwave absorption performance, thus it can be ignored.^37^

## 3. Results and discussion

### 3.1. Preparation of Fe_3_O_4_/HNT-PS nanoabsorbent

**Scheme 2:**
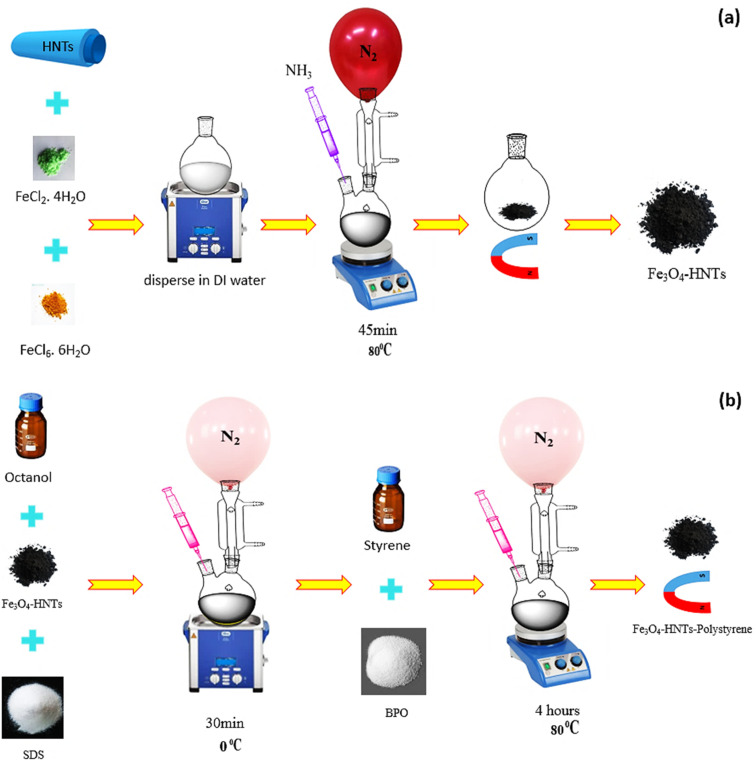
Schematic presentation of the preparation route of (a) Fe_3_O_4_/HNT particles, and (b) Fe_3_O_4_/HNT-PS absorbent system.

As previously discussed, several types of the materials with different structures can be applied for absorbing microwaves. In this study, iron oxide nanoparticles (Fe_3_O_4_ NPs) and halloysite nanotubes (HNTs) were used in composition with polystyrene (PS). The main target of this composition was to limit the absorption mechanisms to only magnetic and dielectric loss due to non-conductivity of the PS. Herein, co-precipitation of the iron salts (Fe^2+^ and Fe^3+^) and further polymerization of the PS through an *in situ* process were considered for construction of an stable composite.^66,67^Scheme 2 schematically represents the preparation route of Fe_3_O_4_/HNT-PS nanoabsorbent. According to the scheme, HNTs were dispersed water *via* ultrasonication, and then iron salts were added into the mixture of HNTs. Co-precipitation of the Fe^2+^ and Fe^3+^ ions occurred through addition of ammonia solution through a dropwise manner. Nitrogen gas was purged into the reaction flask because oxidation by oxygen in the air may lead to the formation of γ-Fe_2_O_3_ NPs, which are less magnetic than the Fe_3_O_4_ NPs.^68–70^ Appearance of black particles confirmed successful preparation of Fe_3_O_4_/HNT nanoparticles. Octanol and sodium dodecyl were dissolved in water, and added to the dispersed Fe_3_O_4_/HNT particles. Afterward, styrene and benzoyl peroxide were added. At this stage, an *in situ* polymerization was performed by sonication (50 kHz, 100 W L^−1^) at 0 °C under the nitrogen flow. Finally, the prepared Fe_3_O_4_/HNT-PS particles were magnetically separated and dried. Afterward, microwave nanoabsorbents were prepared as follows: for the single-layer structure, a specific amount of Fe_3_O_4_/HNT-PS nanocomposite (turned into a powder after drying), was combined with the desired amount of epoxy resin and poured into the mold. For the bilayer structure, the single-layer absorbent was prepared twice in a thinner thickness with the epoxy resin between two layers (thickness reached 1.0 mm). These prepared samples were finally subjected to the microwave radiation.

### 3.2. Characterization

#### 3.2.1. FTIR analysis

**Fig. 1:**
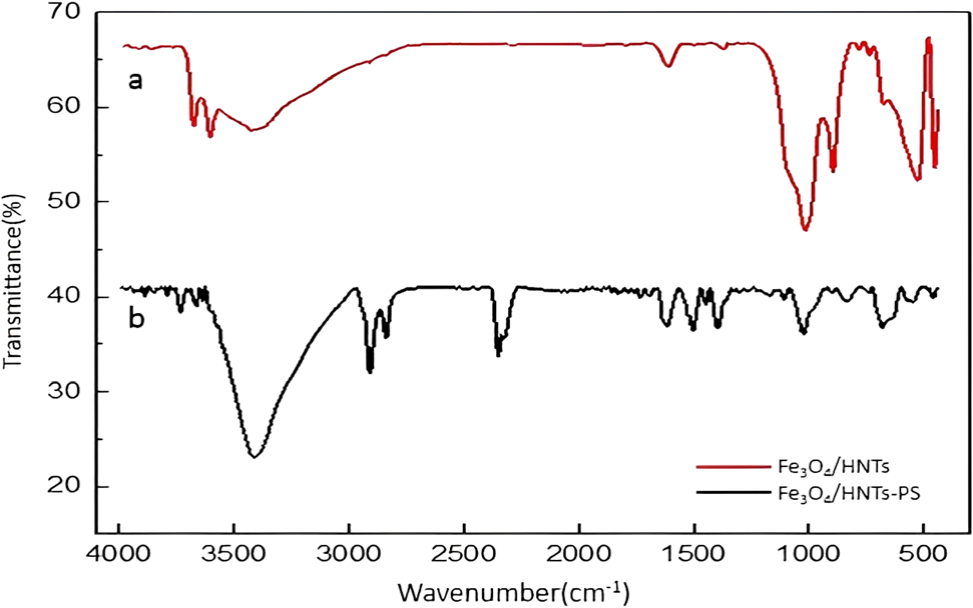
FTIR spectra of the prepared (a) Fe_3_O_4_/HNT particles, and (b) Fe_3_O_4_/HNT-PS absorbent system.

Fourier-transform infrared (FTIR) spectroscopy is one of the common methods to identify and determine the structural characteristics of the prepared compounds. The FTIR spectra of Fe_3_O_4_/HNT and Fe_3_O_4_/HNT-PS are illustrated in Fig. 1. The comparisons can confirm successful preparation of Fe_3_O_4_/HNT-PS nanocomposite. As can be seen in provided spectrum of Fe_3_O_4_/HNT, the bands at 3695 and 3627 cm^−1^ are attributed to the stretching vibrations of inner-surface Al–OH.^41^ The peaks related to the bending vibrations of Al–O–Si and Si–O–Si bands appeared at 536 and 468 cm^−1^, respectively.^71,72^ The strong band around 1046 cm^−1^ can be assigned to Si–O stretching vibration.^73^ In addition, the Fe_3_O_4_ characteristic peak at around 550 cm^−1^ overlapped with the peak related to the bending vibrations of Al–O–Si. As shown in the spectrum of Fe_3_O_4_/HNT-PS, the main characteristic peaks of HNTs are clearly seen. Also, the peaks at the areas 1441, 1516, and 1624 cm^−1^ are related to the C=C bond, present in the structure of styrene.^74,75^ Furthermore, the peak related to the stretching vibrations of C–H bonds appeared at *ca.* 2914 and 3000 cm^−1^.^76^

#### 3.2.2. FESEM analysis

**Fig. 2:**
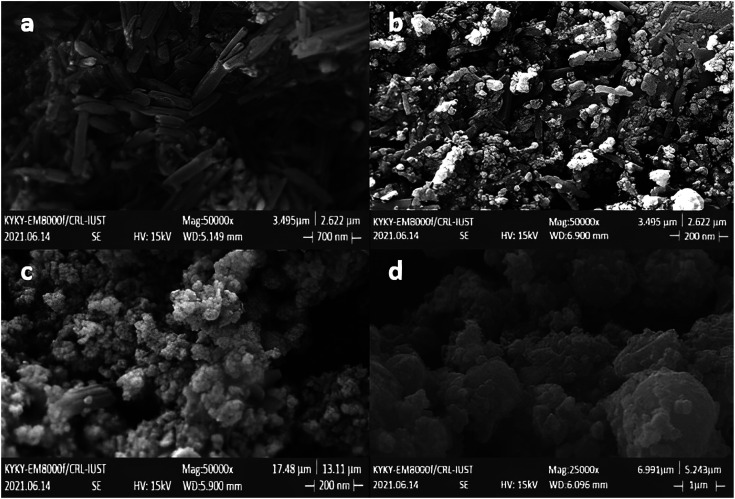
FESEM images of the prepared samples: (a) HNTs, (b) Fe_3_O_4_/HNTs, and (c, d) Fe_3_O_4_/HNT-PS absorbent system.

The morphology and size of the prepared Fe_3_O_4_ nanoparticles, Fe_3_O_4_/HNT nanoparticles and Fe_3_O_4_/HNT-PS nanocomposite were monitored with field-emission scanning-electron microscopy (FESEM). The obtained images confirmed loading of Fe_3_O_4_ and PS on HNTs surfaces (Fig. 2). The tubular structures visible in Fig. 2a are assigned to halloysite nanotubes with outer diameter of 40 to 70 nm. Also, the length of these tubes is estimated to be in a range of 100–2000 nm. The spherical particles of Fe_3_O_4_ clumped on the surface of HNTs with the average size of 60–70 nm are clearly discerned in Fig. 2b. The cluster-shapes formed by the spheres is attributed to the magnetic interactions between the particles. As is observed, the located Fe_3_O_4_ NPs onto the HNTs surfaces caused a roughness in the structure. In Fig. 2c, combination of the PS network has led to a lump-like structure showing non-uniform PS-coating on the magnetized HNTs. Fig. 2c and d illustrate that integration of the particles by the PS network created a porous lattice of the Fe_3_O_4_/HNT. Such porous lattices are suitable for the attenuation of the electromagnetic waves through absorption once the microwaves get into the close spaces.

#### 3.2.3. EDX analysis

**Fig. 3:**
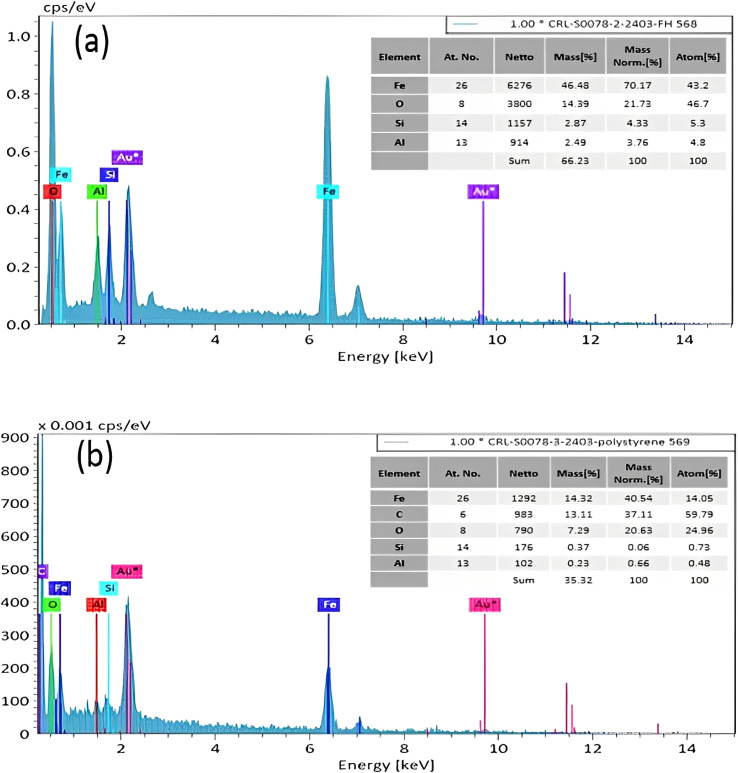
EDX analyses of the (a) Fe_3_O_4_/HNTs, and (b) Fe_3_O_4_/HNT-PS absorbent system.

Energy-dispersive X-ray (EDX) analysis demonstrates the present elements in the structure of the prepared nanocomposite. The obtained results indicated that Fe_3_O_4_/HNT contains Fe, O, Al, and Si elements (Fig. 3a) their exact amounts are seen in related quantitative results. In addition, the presence of carbon atom is notable in Fe_3_O_4_/HNT-PS nanocomposite, confirming successful combination of PS with the Fe_3_O_4_/HNT structure that contains Fe, O, Al, Si and C elements (Fig. 3b). It should be noted that the quantitative values presented in Fig. 3 well verified the presence of the desired atoms in the compounds and also indicated the absence of impurities in the compound. It is noteworthy that the Au impurities is due to the sample preparation process for EDX and FESEM analyses. The purity of the prepared nanocomposite was confirmed by the absence of other elements.

#### 3.2.4. Vibrating-sample magnetometer (VSM)

**Fig. 4:**
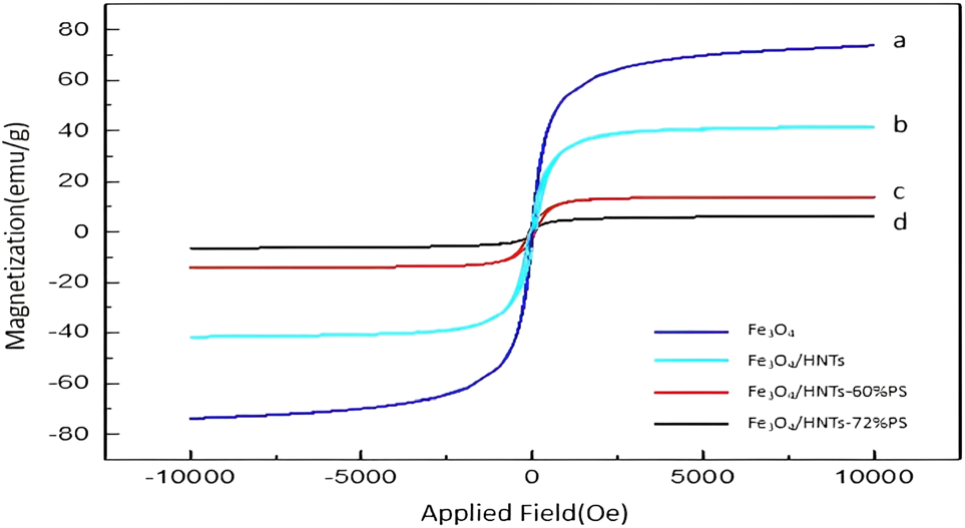
Magnetization curves of the synthesized samples: (a) Fe_3_O_4_, (b) Fe_3_O_4_/HNT, (c) Fe_3_O_4_/HNT-60% PS and (d) Fe_3_O_4_/HNT-72% PS absorbent system.

According to literature, a super-paramagnetic behavior is seen for the neat Fe_3_O_4_ NPs. This unique property exclusively belongs to the nanomaterials (below 50 nm), which are not able to form a double-domain magnetization state due to their tiny scale.^77–79^ The results of saturation of magnetization in prepared Fe_3_O_4_/HNT-PS nanocomposite is displayed in Fig. 4. As can be seen, the Fe_3_O_4_/HNT-PS nanocomposite has shown thin hysteresis VSM loops, so it can be classified as a soft magnetic material. Therefore, the Fe_3_O_4_/HNT-PS nanocomposite can be very easily magnetized through applying an external magnetic field, and the magnetism is lost upon removing that. This issue is also true for the iron oxide magnetic nanoparticles, which means that it can be classified as a soft ferromagnetic species due to its thin hysteresis VSM loops.^37^ In the hysteresis loops, the super-paramagnetic behavior of the Fe_3_O_4_ can be visible in the VSM analysis.^80,81^ Also, the magnetic saturation value of the prepared samples were decreased after incorporation of non-magnetic materials (like HNTs and PS). It is quite reasonable to see that the magnetic saturation values for Fe_3_O_4_, Fe_3_O_4_/HNT, Fe_3_O_4_/HNT-60% PS and Fe_3_O_4_/HNT-72% PS are 73.8, 41.6, 14 and 6.5 emu g^−1^, respectively.

#### 3.2.5. TGA

**Fig. 5:**
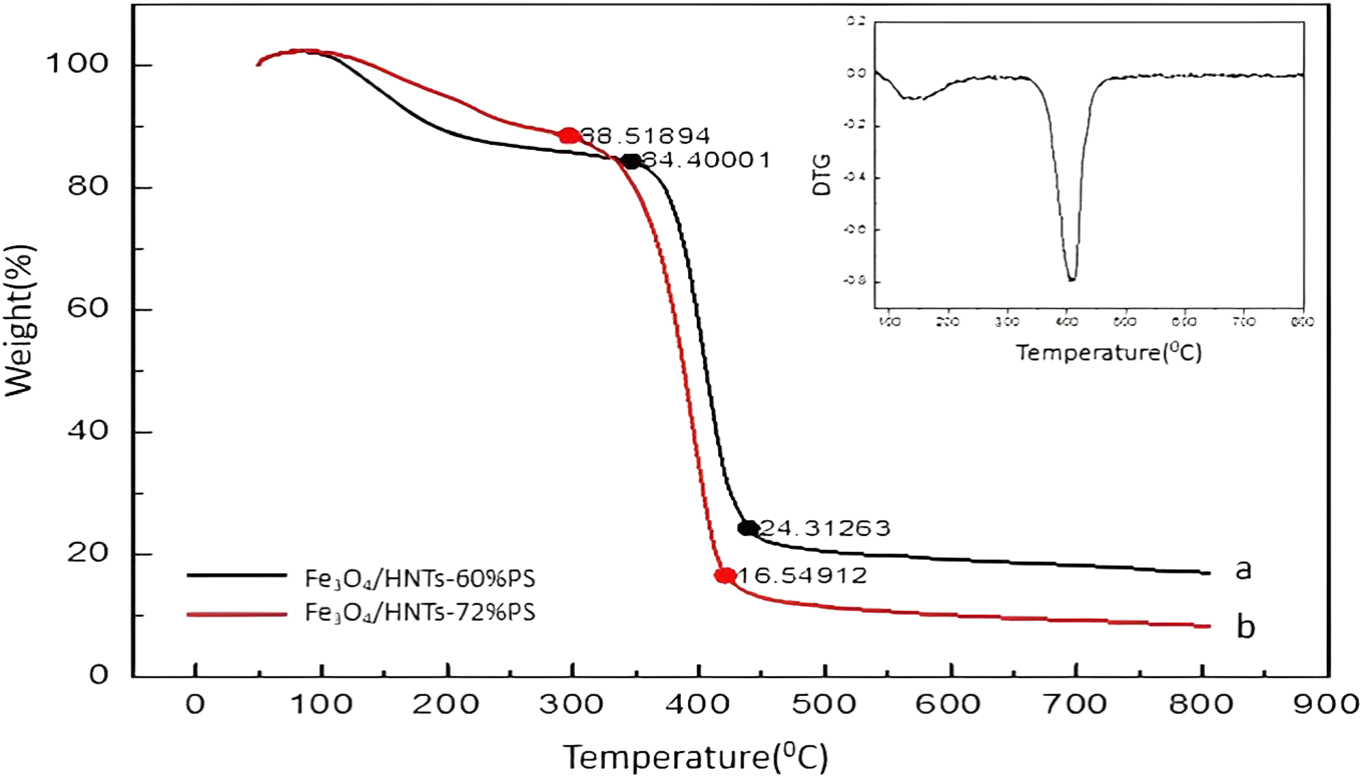
The TGA analyses of the synthesized samples: (a) Fe_3_O_4_/HNTs-60% PS and (b) Fe_3_O_4_/HNTs-72% PS absorbent systems.

The thermogravimetric analysis (TGA) and derivative thermogravimetric (DTG) results provide comprehensive information about the sample's thermal stability. Based on this method, thermal degradation trend of the Fe_3_O_4_/HNT-PS nanocomposite is shown in Fig. 5. The reason for preparation of different weight percentages is to investigate the effect of the amount of each material which are used for the reflection loss. Therefore, TGA analysis will be useful to determine the amount of polymer by increasing the temperature of the prepared samples. As is observed, a 15% weight loss at the initial stage (in a range of 100–300 °C) is related to removal of the entrapped water molecules in silica layer, and also the adsorbed moisture.^82^ The Fe_3_O_4_/HNT-PS nanocomposite with the different weight percentage of polystyrene (60% and 72%) shown a significant weight loss in a thermal range of *ca.* 300 to 450 °C, which can be attributed to decomposition of the PS structure.^83^ According to DTG curve, a peak at about 410 °C indicates the highest mass change rate. Afterward, a fairly gradual degradation trend is seen at the temperature above 500 °C, which is related to degradation of Fe_3_O_4_ and HNTs.^53^ The Fe_3_O_4_ nanoparticles have good thermal stability up to 750 °C.^84^ Also, dehydroxylation of halloysite nanotube occurred at 480–640 °C.^85^ Based on these results, it can be concluded that the architecture of the Fe_3_O_4_/HNT-PS nanocomposite is stable enough to be used in the wave-absorption process for several times. In addition to above analyses, X-ray diffraction (XRD) pattern, SEM, and dynamic light scattering (DLS) curve of the neat Fe_3_O_4_ NPs were also provided for better comparison with the Fe_3_O_4_/HNTs-72% PS absorbent systems (Fig. S1–S3, in ESI file†).

### 3.3. Microwave absorption properties

#### 3.3.1. Vector network analysis

**Fig. 6:**
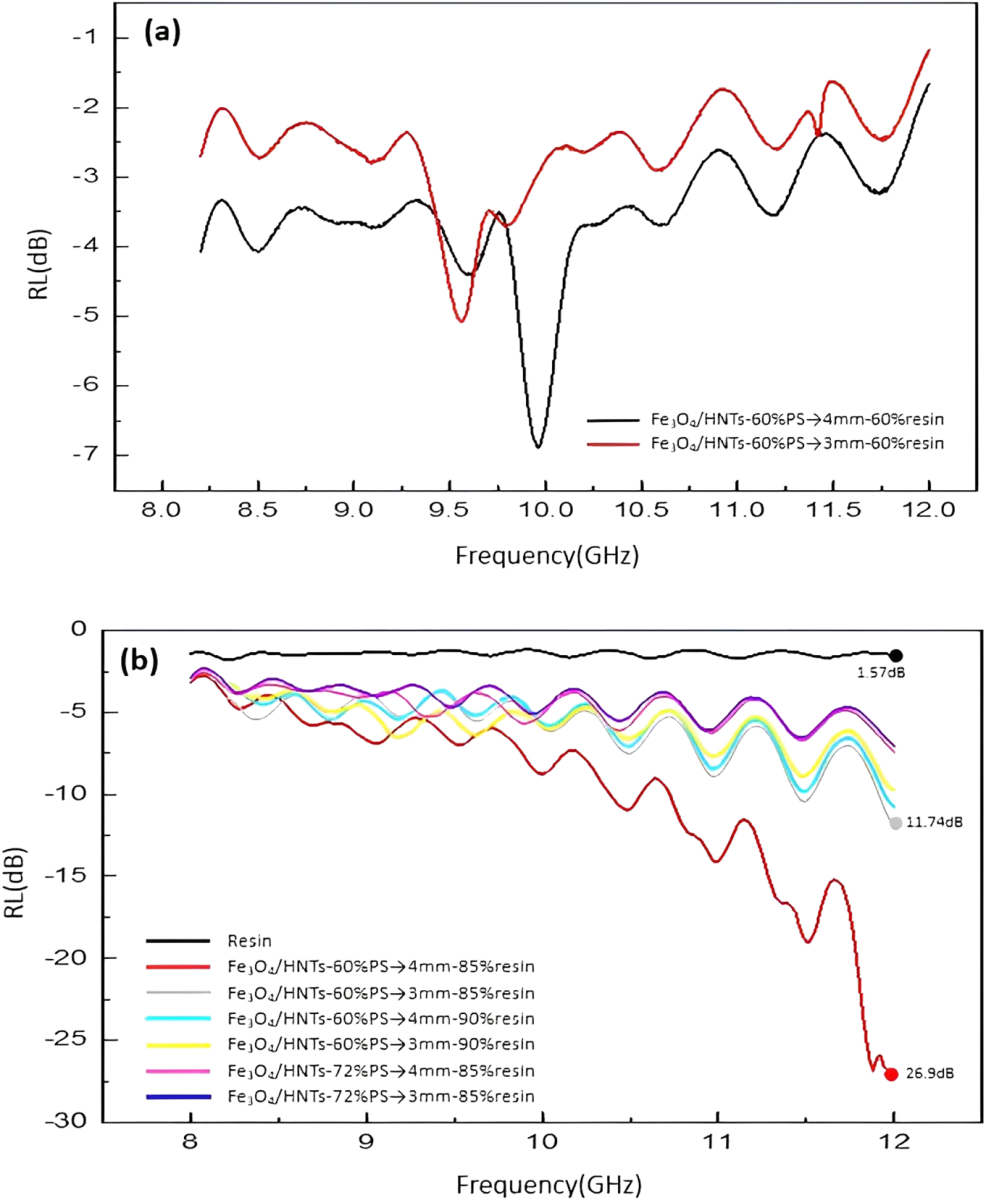
Frequency dependence of the calculated RL values of the Fe_3_O_4_/HNT-PS single-layer samples (a) and Fe_3_O_4_/HNT-PS bilayer samples (b).

**Scheme 3:**
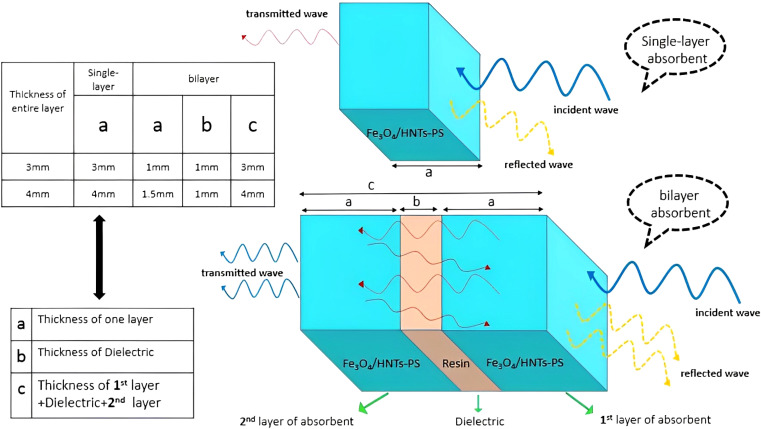
Schematic presentation of the single-layer and bilayer absorbent thicknesses.

The microwave absorption spectra of the samples were provided by vector network analysis (VNA), as shown in Fig. 6. Firstly, the effects of resin, various nanocomposite systems, and sample thickness (with different amounts of nanocomposite) were precisely examined on the microwave absorption rate, and the obtained results were summarized in Table 1. Also, the details related to the thickness of the layers are presented in Scheme 3. The reflection loss (RL) of single-layer samples (samples 1 and 4) with the thickness values of 3.0 and 4.0 mm were measured. As is observed, the obtained results with the samples −5.1 dB and −6.9 dB are not so appropriate.^25^ For better absorption, a dielectric layer of resin was incorporated between the layers of Fe_3_O_4_/HNTs-PS composite. In this case, the nanocomposite systems Fe_3_O_4_/HNT-60% PS and Fe_3_O_4_/HNT-72% PS with a thickness value of 3.0 mm were prepared and tested (samples 2 and 7). The Fe_3_O_4_/HNT-60% PS nanocomposite with RL-11.74 dB exhibited better microwave absorption activity in comparison with the Fe_3_O_4_/HNT-72% PS system, which can be the result of using a smaller amount of a non-conductive polymer (polystyrene). As can be seen in Table 1, sample 2 containing 85 wt% of resin had a higher amount of microwave absorption with the fixed thickness of 3.0 mm. So, the sample with 85% resin was chosen as the best absorbent. In the same line, the Fe_3_O_4_/HNT-60% PS nanocomposite with 85% of resin exhibited an acceptable result, as well. The effect of the absorbent's amount using the sample with 4.0 mm was investigated, too. As presented in Table 1, sample 5 was chosen as the most efficient sample with an RL value of −26.9 dB. In fact, application of very thin layers of resin (as a dielectric material) leads to entrapment of the absorbed microwaves, as can be seen in the red simulated waves in Scheme 1. Also, as it was mentioned in its description, incorporation of a dielectric material with different physical properties between the layers of the absorbent is the main reason for efficient capture of the waves.^86,87^ Thus, increase in the microwave absorption is approved by sinusoidal shape of the resin diagram. Also, this feature was confirmed by microwave absorption curve (Fig. 6b), where the sinusoidal diagram is the best approval for entrapping the waves between two layers of the absorbent system. As can be seen in Fig. 6b, the absorption intensity increased by decreasing the weight percentage of the polymer due to the enhanced magnetization intensity. Also, it was observed that proportional to increasing the weight ratio of resin, the absorption rate is significantly decreased. Besides, the absorption rate was improved by increasing the thickness from 3.0 to 4.0 mm. It should be noticed that the sinusoidal shape can disclose the reason of attenuation of the wave's energy. The presence of a dielectric layer causes the microwaves to go back and forth between two absorbent layers, resulting in the change of the essence of the waves from high frequencies to lower levels.

**Table 1:** Reflection loss values that measured and details of different samples

Sample	Nanocomposite	Amount of polymer (wt%)	Resin wt%	Thickness (mm)	RL (dB)
1 (single-layer)	Fe_3_O_4_/HNTs-60% PS	60	60	3	−5.07
2 (bilayer)		60	85		−11.74
3 (bilayer)		60	90		−9.71
4 (single-layer)		60	60	4	−6.88
5 (bilayer)		60	85		−26.9
6 (bilayer)		60	90		−10.72
7 (bilayer)	Fe_3_O_4_/HNTs-72% PS	72	85	3	−7.05
8 (bilayer)		72	85	4	−7.4
Pure resin	—	0	100	3	−1.7

**Fig. 7:**
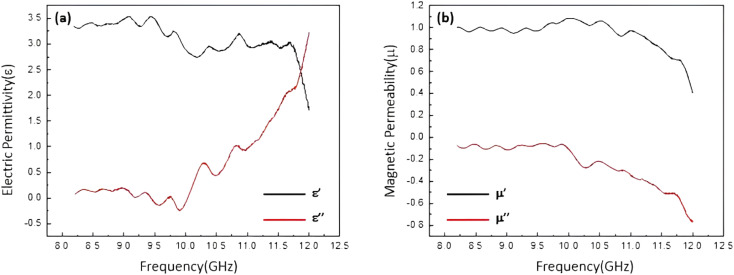
Frequency dependent of the complex permittivity (a) and complex permeability (b) of the best sample: Fe_3_O_4_/HNT-60% PS with the thickness of 4.0 mm and 85% resin of the pellet.

The electrical permittivity and magnetic permeability values related to sample 5 (the most efficient one) are presented in Fig. 7. According to literature, the microwave absorption performance of an absorbent system is closely in correlation with the complex permittivity (*ε*_r_) and complex permeability (*μ*_r_) parameters. Therefore, to emphasize that the absorption properties of a conventional absorbent is related to the electrical and magnetic properties of the involved components, electric permittivity and magnetic permeability diagrams were obtained for the prepared samples. Fig. 7a represents the complex electric permittivity as a function of frequency, where the amount of *ε*′ is decreasing from 1.5 to 3.5, and the amount of *ε*′′ is approximately from 0 to 3.25. It can be seen that proportional to increase in the frequency value from 8 to 12 GHz, the amount of *ε*′′ (which indicates the amount of electromagnetic energy) has lost. In fact, this amount of energy loss is converted into the heat energy.^12^ As presented in Fig. 7a, *ε*′′ is also consistent with the graph of reflection loss. Also, Fig. 7b demonstrates dependence of the frequency on *μ*′ and *μ*′′ values of the samples. *μ*′ and *μ*′′ are the imaginary and real parts of magnetic permeability that will change due to the change of the environment and the entry of microwaves into the secondary environment which has different dielectric properties.^14^ It is observed that the values of *μ*′ and *μ*′′ are changed according to the frequency variation. The negative values of *μ*′′ of the magnetic permeability (which indicates energy dissipation) can also be examined according to Wu's theory,^88^ which suggests that the negative values of *μ*′′ are ascribed to the induced magnetic energy, going out of the microwave absorbing materials. As presented in the figure, the percentage of the absorption obtained for the most efficient sample is *ca.* 95% of the radiated microwave.

#### 3.3.2. Comparison of previous similar single-layer and bilayer systems with Fe_3_O_4_/HNT-PS

Table 2 briefly presents a survey of various types of microwave absorbent systems with almost similar compositions. It should be noticed that there would be differences between the compared systems such as utilization of two different layers in bilayer types, and the absence of dielectric between the absorbent layers. Most absorbent systems show acceptable results of microwave absorption (materials are selected based on it in studies), which are a combination of metals, magnetic nanoparticles, ferrites with carbon-based conductive polymers.^14^ In bilayer absorbent systems, it is expected that due to the presence of two absorbent layers and microwave collision, energy attenuation of the microwave occurs more than that of a single-layer absorbent, meaning that the value of RL is higher than the single-layer absorbent. Of course, the amount of absorption also depends on the thickness of the layers. Hence, it is reasonable to that a single-layer absorbent increases the amount of RL by increasing the thickness of the layer, but it does not mean that the optimal result will be obtained.^37^ The materials selected in the current work were chosen according to the investigation of the expected results to understand how the PS (as a non-conducting polymer) affects the absorption results, or what would be the effects of a bilayer absorbent with the presence of a dielectric between the layers. For better understanding of this issue, we can take two opposite mirrors as an example (at the frequency of visible light) where the light is trapped between them and constantly radiates to the opposite side, and as a result, we will heat up the surface of the mirror and absorb energy by the surface of the mirror. Comparisons made in Table 2 would be useful to quickly figure out the effectiveness of different ingredients and the architecture on the absorption results.

**Table 2:** Comparison of single-layer and bilayer microwave absorbents with the present research

Materials	Thickness/mm	RL/dB	Frequency/GHz	Bandwidth (≤10 dB)/GHz	Ref.
PS-PVP-Fe_3_O_4_- PEG (one layer)	1	−11	10	0.3	89
PS/TGO/Fe_3_O_4_ (one layer)	—	−35	11.9	4	90
Ni@MWCNT/PS (one layer)	6	−33	2.7	0.9	91
HNTs/polypyrrole/Fe_3_O_4_ (one layer)	3	−31.2	10.58	4	37
HNTs/Fe_3_O_4_/C (one layer)	4.56	−51.5	11	—	92
PBOPy/PPy/Fe_3_O_4_ (one layer)	3.5	−23.3	13	2.2	93
PEDOT/PSS/HNTs (one layer)	4.5	−16.3	8.4	2	94
Fe3O4/carbon@Fe3O4/rGO (bilayer)	2	−52	9.4	4	95
LAS@LAS-SiC (bilayer)	4	−42.8	10.5	3.5	96
Fe_3_O_4_/HNT-PS (current work)	4	−26.9	12	1.27	—

## 4. Conclusion

In this work, the prepared Fe_3_O_4_/HNTs-PS magnetic nanocomposite was investigated as a microwave absorbent system in the X-band region. Firstly, the presented system and its exclusive properties have been studied *via* various methods such as FTIR, TGA, EDX, VSM and FESEM. Then, the effects of magnetic and dielectric dissipative agents on the absorption process have been examined by applying polystyrene (PS), as a non-conductive polymer. Totally, three main factors affect the microwave absorption: magnetic loss, dielectric loss and conduction loss. In this research, the intention was to limit one of the mentioned factors and investigate its effectiveness. For this purpose, polystyrene (PS, as a non-conductive polymer) has been used to quench the conduction loss, and play a role in integrating other components. The observed significant reduction of RL confirmed the effect of non-conductive PS. Indeed, the use of PS (which is a non-conductive agent) has decreased the amount of microwave energy attenuation by limiting the condition of conduction loss, which is one of the most effective factors in microwave absorption. Therefore, the microwave absorption has diminuend trough incorporation of the PS into the structure. In addition, it was observed that the incorporation of a dielectric material between two layers of the absorbent material enhances the microwave absorption *via* entrapment of the waves between the layers. According to the obtained results, the absorption value for a single-layer absorbent system with a thickness of 4.0 mm is reduced by −20.02 dB through using a bilayer analogue. It has been corroborated that when the electromagnetic wave enters into the absorbent system, the waves between the different layers of the absorbent acts back and forth due to the presence of a dielectric material between the layers. Therefore, a considerable absorption value has been reached by losing the wave's energy. The thickness of the layers, the PS polymer, and weight percentages of the used resin were optimized in this work. Concisely, it has been observed that the best result (95% of wave's energy, −26.9 dB at 12 GHz) achieves by using a bilayer Fe_3_O_4_/HNTs-PS sample with the thickness of 4.0 mm, containing 85% of the dielectric resin and 60 wt% of the PS. Ultimately, the presented absorbent system is suggested to be scaled up for the industrial exploitation due to the low-cost raw materials, convenience of the preparation method, high performance, and also the use of multilayer absorbent by using a dielectric.

## Author contributions

D. F. J. performed bench work; M. R. gave the hypothesis, provided the analyses and interpreted them, and also prepared the initial draft; R. T. L. reviewed and edited the context, revised, and improved the whole document; Z. H. participated in conceptualization and practical sections; A. M. led the whole project as the supervisor and project administrator.

## Conflicts of interest

The authors declare no conflict of interest.

## Supplementary Material

RA-013-D2RA08339F-s001

## References

[cit1] Chung D. D. L. (2012). Carbon.

[cit2] Kong L. B., Li Z. W., Liu L., Huang R., Abshinova M., Yang Z. H., Tang C. B., Tan P. K., Deng C. R., Matitsine S. (2013). Int. Mater. Rev..

[cit3] Chen J., Li Y., Huang L., Li C., Shi G. (2015). Carbon.

[cit4] Anooja J. B., Dijith K. S., Surendran K. P., Subodh G. (2019). J. Alloys Compd..

[cit5] Hosseini H., Mahdavi H. (2018). Appl. Organomet. Chem..

[cit6] Najim M., Puthucheri S., Agarwala V., Singh D. (2015). Mater. Sci. Mater..

[cit7] Tian C., Du Y., Xu P., Qiang R., Wang Y., Ding D., Xue J., Ma J., Zhao H., Han X. (2015). ACS Appl. Mater. Interfaces.

[cit8] Vidhya L., Subodh G. (2022). J. Mater. Chem. C.

[cit9] Zhang S., Jia Q., Zhao Y., Li H., Wu Q. (2014). J. Mater. Chem. C.

[cit10] Heng L., Zhang Z., Chen X., Wang S., Wu Z., Xie Z., Tang Z., Zou Y. (2019). Appl. Organomet. Chem..

[cit11] Hosseini S. H., Mohseni S. H., Asadnia A., Kerdari H. (2011). J. Alloys Compd..

[cit12] Zhuo R. F., Feng H. T., Chen J. T., Yan D., Feng J. J., Li H. J., Geng B. S., Cheng S., Xu X. Y., Yan P. X. (2008). J. Phys. Chem. C.

[cit13] Lv X., Guo J., Zhao C., Wei Y., Zhang J., Wu Z., Gong C. (2017). Mater. Lett..

[cit14] Rouhi M., Hajizadeh Z., Taheri-Ledari R., Maleki A., Babamoradi M. (2022). J. Mater. Sci. Eng., B..

[cit15] Zhao H., Wang F., Cui L. (2021). Nanomicro Lett..

[cit16] Wang Z., Bi H., Liu J., Sun T., Wu X. (2008). J. Magn. Magn. Mater..

[cit17] Ma Y., Zhou Y., Sun Y., Chen H., Xiong Z., Li X., Shen L., Liu Y. (2019). J. Alloys Compd..

[cit18] Liu X., Guo H., Xie Q., Luo Q., Wang L., Peng D. (2015). J. Alloys Compd..

[cit19] Xu P., Han X. J., Liu X. R., Zhang B., Wang C., Wang X. H. (2009). J. Mater. Chem..

[cit20] Lv R., Kang F., Gu J., Gui X., Wei J., Wang K., Wu D. (2008). Appl. Phys. Lett..

[cit21] Zeng X., Cheng X., Yu R., Stucky G. D. (2020). Carbon.

[cit22] Liu J., Feng Y., Qiu T. (2011). J. Magn. Magn. Mater..

[cit23] Lu B., Dong X. L., Huang H., Zhang X. F., Zhu X. G., Lei J. P., Sun J. P. (2008). J. Magn. Magn. Mater..

[cit24] Liu J. R., Itoh M., Machida K. (2003). Appl. Phys. Lett..

[cit25] Zhang X. F., Dong X. L., Huang H., Liu Y. Y., Wang W. N., Zhu X. G., Lv B., Lei G. P. (2006). Appl. Phys. Lett..

[cit26] Liu X. G., Geng D. Y., Meng H., Shang P. J., Zhang Z. D. (2008). Appl. Phys. Lett..

[cit27] Wen F., Zhang F., Liu Z. (2011). J. Phys. Chem. C.

[cit28] Huo J., Wang L., Yu H. (2009). J. Mater. Sci..

[cit29] Tung T. T., Feller J. F., Kim T., Kim H., Yang W. S., Suh K. S. (2011). J. Polym. Sci..

[cit30] Wei J., Liu J., Li S. (2007). J. Magn. Magn. Mater..

[cit31] Zhao R., Jia K., Wei J., Pu J.-X., Liu X. B. (2010). Mater. Lett..

[cit32] Peymanfar R., Norouzi F., Javanshir S. (2019). Synth. Met..

[cit33] Saini P., Choudhary V., Singh B. P., Mathur R. B., Dhawan S. K. (2011). Synth. Met..

[cit34] Hosseini S. H., Entezami A. A. (2003). J. Appl. Polym. Sci..

[cit35] Heidari B., Ansari M., Hoseinabadi A., Jiriaee H., Heidary F. (2016). J. Mater. Sci. Mater. Electron.

[cit36] Jaleh B., Madad M. S., Tabrizi M. F., Habibi S., Golbedaghi R., Keymanesh M. R. (2011). J. Iran. Chem. Soc..

[cit37] Maleki S. T., Babamoradi M., Rouhi M., Maleki A., Hajizadeh Z. (2022). Synth. Met..

[cit38] Maleki A., Taheri-Ledari R., Ghalavand R., Firouzi-Haji R. (2020). J. Phys. Chem. Solids.

[cit39] Rahimi J., Taheri-Ledari R., Niksefat M., Maleki A. (2020). Catal. Commun..

[cit40] Vidhya L., Subodh G. (2020). J. Electron. Mater..

[cit41] Rouhi M., Babamoradi M., Hajizadeh Z., Maleki S. T., Maleki A. (2020). Optik.

[cit42] Qin H., Wang C. M., Dong Q. Q., Zhang L., Zhang X., Ma Z. Y., Han Q. R. (2015). J. Magn. Magn. Mater..

[cit43] Zhou L., Pan S., Chen X., Zhao Y., Zou B., Jin M. (2014). J. Chem. Eng..

[cit44] Noh J., Osman O. I., Aziz S. G., Winget P., Brédas J. L. (2015). J. Mater. Chem..

[cit45] Xi G., Yue B., Cao J., Ye J. (2011). Eur. J. Chem..

[cit46] Zhu H.-Y., Fu Y. Q., Jiang R., Jiang J.-H., Xiao L., Zeng G. M., Zhao S. L., Wang Y. (2011). J. Chem. Eng..

[cit47] Chen X., Zhou Y., Han H., Wang X., Zhou L., Yi Z., Fu Z., Wu X., Li G., Zeng L. (2021). Mater. Today Chem..

[cit48] Ahmad S., Riaz U., Kaushik A., Alam J. (2009). J. Inorg. Organomet. Polym..

[cit49] Ma C., Li C., He N., Wang F., Ma N., Zhang L., Lu Z., Ali Z., Xi Z., Li X., Liang G., Liu H., Deng Y., Xu L., Wang Z., Biomed J. (2012). Nanotech.

[cit50] Huang X., Lu M., Zhang X., Wen G., Zhou Y., Fei L. (2012). Scr. Mater..

[cit51] Zhou W., Hu X., Bai X., Zhou S., Sun C., Yan J., Chen P. (2011). ACS Appl. Mater. Interfaces.

[cit52] Li X., Zhang B., Ju C., Han X., Du Y., Xu P. (2011). J. Phys. Chem. C.

[cit53] Maleki S. T., Babamoradi M., Hajizadeh Z., Maleki A., Rouhi M. (2020). Micro Nano Lett..

[cit54] Taheri-Ledari R., Ahghari M. R., Ansari F., Forouzandeh-Malati M., Mirmohammadi S. S., Zarei-Shokat S., Ramezanpour S., Zhang W., Tian Y., Maleki A. (2022). Nanoscale Adv..

[cit55] Maleki A., Hajizadeh Z., Sharifi V., Emdadi Z. (2019). J. Clean. Prod..

[cit56] Jian X., Wu B., Wei Y., Dou S. X., Wang X., He W., Mahmood N. (2016). ACS Appl. Mater. Interfaces.

[cit57] Zhang W., Taheri-Ledari R., Hajizadeh Z., Zolfaghari E., Ahghari M. R., Maleki A., Hamblin M. R., Tian Y. (2020). Nanoscale.

[cit58] Maleki A., Taheri-Ledari R., Rahimi J., Soroushnejad M., Hajizadeh Z. (2019). ACS Omega.

[cit59] Maleki A., Taheri-Ledari R., Soroushnejad M. (2018). ChemistrySelect.

[cit60] Taheri-Ledari R., Rahimi J., Maleki A. (2019). Ultrason. Sonochem..

[cit61] Taheri-Ledari R., Zhang W., Radmanesh M., Mirmohammadi S. S., Maleki A., Cathcart N., Kitaev V. (2020). Small.

[cit62] Taheri-Ledari R., Zolfaghari E., Zarei-Shokat S., Kashtiaray A., Maleki A. (2022). Commun. Biol..

[cit63] Kassaee M. Z., Motamedi E., Majdi M. (2011). J. Chem. Eng..

[cit64] Xiang C., Pan Y., Guo J. (2007). Ceram. Int..

[cit65] Feng Y. B., Qiu T., Shen C. Y. (2007). J. Magn. Magn. Mater..

[cit66] Forouzandeh-Malati M., Ganjali F., Zamiri E., Zarei-Shokat S., Jalali F., Padervand M., Taheri-Ledari R., Maleki A. (2022). Langmuir.

[cit67] Taheri-Ledari R., Tarinsun N., Sadat Qazi F., Heidari L., Saeidirad M., Ganjali F., Ansari F., Hassanzadeh-Afruzi F., Maleki A. (2023). Inorg. Chem..

[cit68] Taheri-Ledari R., Maleki A. (2021). New J. Chem..

[cit69] Soltani S. S., Taheri-Ledari R., Farnia S. M. F., Maleki A., Foroumadi A. (2020). RSC Adv..

[cit70] Taheri-Ledari R., Rahimi J., Maleki A., Esmail Shalan A. (2020). New J. Chem..

[cit71] Taheri-Ledari R., Zhang W., Radmanesh M., Cathcart N., Maleki A., Kitaev V. (2021). J. Nanobiotechnology.

[cit72] Hajizadeh Z., Valadi K., Taheri-Ledari R., Maleki A. (2020). ChemistrySelect.

[cit73] Hajizadeh Z., Hassanzadeh-Afruzi F., Jelodar D. F., Ahghari M. R., Maleki A. (2020). RSC Adv..

[cit74] Buruga K., Kalathi J. T., Kim K.-H., Ok Y. S., Danil B. (2018). J. Ind. Eng. Chem..

[cit75] Taheri-Ledari R., Maleki A., Zolfaghari E., Radmanesh M., Rabbani h., Salimi A., Fazel R. (2020). Ultrason. Sonochem..

[cit76] Taheri-Ledari R., Maleki A. (2020). J. Pept. Sci..

[cit77] Taheri-Ledari R., Rasouli Asl F., Saeidirad M., Kashtiaray A., Maleki A. (2022). Sci. Rep..

[cit78] Taheri-Ledari R., Sadat Qazi F., Saeidirad M., Maleki A. (2022). Sci. Rep..

[cit79] Ganjali F., Kashtiaray A., Zarei-Shokat S., Taheri-Ledari R., Maleki A. (2022). Nanoscale Adv..

[cit80] Maleki A., Taheri-Ledari R., Ghalavand R. (2020). Comb. Chem. High Throughput Screen..

[cit81] Taheri-Ledari R., Hashemi S. M., Maleki A. (2019). RSC Adv..

[cit82] Taheri-Ledari R., Mirmohammadi S. S., Valadi K., Maleki A., Shalan A. E. (2020). RSC Adv..

[cit83] Ding P., Qu B. (2005). J. Colloid Sci..

[cit84] Kadi S., Lellou S., Marouf-Khelifa K., Schott J., Gener-Batonneau I., Khelifa A. (2012). Microporous Mesoporous Mater..

[cit85] Chen J., Feng J., Yan W. (2016). J. Colloid Sci..

[cit86] Danlée Y., Huynen I., Bailly C. (2012). Appl. Phys. Lett..

[cit87] Oikonomou A., Giannakopoulou T., Litsardakis G. (2007). J. Magn. Magn. Mater..

[cit88] Wu H., Wang L., Wang Y., Guo S., Shen Z. (2012). J. Alloys Compd..

[cit89] Hosseini S. H., Sadeghi M. (2014). Curr. Appl. Phys..

[cit90] Chen Y., Wang Y., Zhang H.-B., Li X., Gui C.-X., Yu Z.-Z. (2015). Carbon.

[cit91] Srivastava R. K., Narayanan T. N., Reena Mary A. P., Anantharaman M. R., Srivastava A., Vajtai R., Ajayan P. M. (2011). Appl. Phys. Lett..

[cit92] Kaixuan S., Zilong H., Shupei L., Jing O. (2022). Appl. Clay Sci..

[cit93] Li Y., Chen D., Liu X., Zhou Y., Zhuang Q., Cai R., Zhang K. (2014). Compos. Sci. Technol..

[cit94] Luo S. J., Zhang P., Mei Y. A., Chang J. B., Yan H. (2016). J. Appl. Polym. Sci..

[cit95] Gang Q., Niaz Akhtar M., Boudaghi R. (2021). J. Magn. Magn. Mater..

[cit96] Peng C.-H., Shiu Chen P., Chang C.-C. (2014). Ceram. Int..

