# Supplementary material for: A magnetic X-band frequency microwave nanoabsorbent made of iron oxide/halloysite nanostructures combined with polystyrene
Source: RSC Adv. 2023 Feb 27;13(10):6643–55. doi: 10.1039/d2ra08339f (PMC9969233; doi:10.1039/d2ra08339f)
Supplement: RA-013-D2RA08339F-s001 [file RA-013-D2RA08339F-s001.pdf]

Electronic Supplementary Material (ESI) for RSC Advances.
This journal is © The Royal Society of Chemistry 2023

A Magnetic X-Band Frequency Microwave Nanoabsorbent Made of Iron Oxide/Halloysite Nanostructures Combined with Polystyrene

Diana Fallah Jelodar[1], Mojtaba Rouhi[2], Reza Taheri-Ledari[1], Zoleikha Hajizadeh[1], Ali Maleki*,[1]

[1]*Catalysts and Organic Synthesis Research Laboratory, Department of Chemistry, Iran University of Science and Technology, Tehran16846-13114, Iran.*

[2]*Department of Physics, Iran University of Science and Technology, Tehran16846-13114, Iran.*

**Corresponding author. (Ali Maleki) E-mail: maleki@iust.ac.ir; Fax: +98-21-73021584; Tel: +98-21-73228313.*

Author's ORCIDs:
Reza Taheri-Ledari: https://orcid.org/0000-0002-6511-9411
Ali Maleki: https://orcid.org/0000-0001-5490-3350

Table of Content

Content	Page
**Figure S1.** XRD pattern of the neat Fe_3O_4 NPs.	*S2*
**Figure S2.** SEM image of the neat Fe_3O_4 NPs.	*S3*
**Figure S3.** DLS curve of the neat Fe_3O_4 NPs.	*S4*

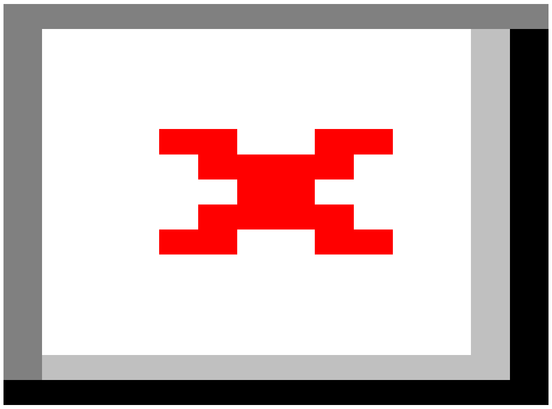

Figure S1. XRD pattern of the neat Fe_3O_4 NPs.

The crystal structure of the neat Fe_3O_4 NPs was studied by the X-ray diffraction (XRD) analysis. It can be seen that the diffraction peaks of the Fe_3O_4 NPs are well confirmed by the reference JCPDS card (NO. 00-024-0072); $2\theta = 33.115^{o}$, 35.612^{o}, 54.005^{o}, 57.508^{o}, 62.385^{o}, 63.966^{o}, and 75.409^{o}.

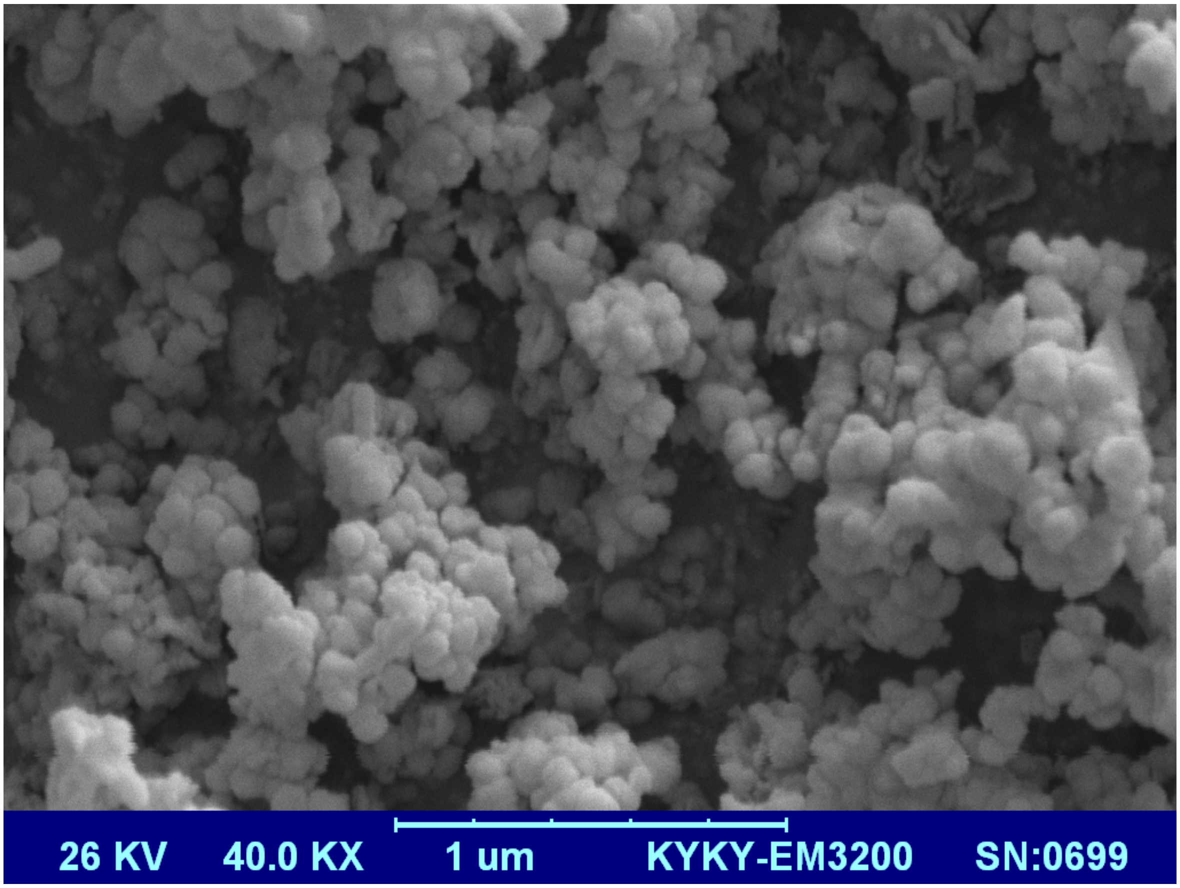

Figure S2. SEM image of the neat Fe_3O_4 NPs.

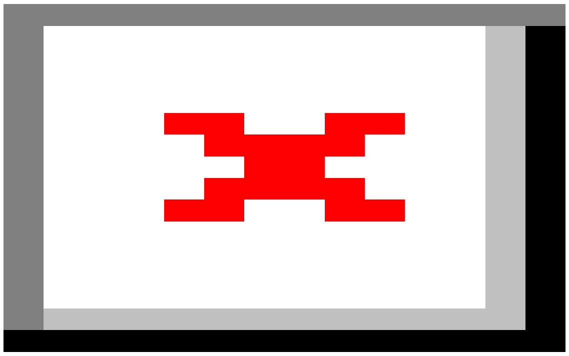

Figure S3. DLS curve of the neat Fe_3O_4 NPs.

Dynamic Light Scattering (DLS) analysis of the neat Fe_3O_4 NPs is shown in Figure S3. Mean particle size in the colloidal state was recorded for this sample is the between 0.04 - 0.1 microns (40-100 nm).
